# Targeting transcription factors in cancer drug discovery

**DOI:** 10.37349/etat.2020.00025

**Published:** 2020-12-28

**Authors:** Partha Mitra

**Affiliations:** Institute of Health and Biomedical Innovation, the Queensland University of Technology, Brisbane 4059, Australia; National University of Singapore, Singapore

**Keywords:** Transcription factor, drug discovery, gene expression, protein-protein interaction

## Abstract

Cancer drug discovery is currently dominated by clinical trials or clinical research. Several potential drug candidates have been brought into the pipeline of drug discovery after showing very promising results at the pre-clinical level and are waiting to be tested in human clinical trials. Interestingly, among the potential drug candidates, a few of them have targeted transcription factors highlighting the fundamental undruggable nature of these molecules. However, using advanced technologies, researchers were recently successful in partly unlocking this undruggable nature, which was considered as a ‘grey area’ in the early days of drug discovery, and as a result, several potential candidates have emerged recently. The purpose of the review is to highlight some of the recently reported studies of targeting transcription factors in cancer and their promising outcomes.

## Introduction

Transcription factors (TFs) are a group of proteins required for gene expression and more precisely in the regulation of gene transcription. In metazoan, they are localized in the nuclear microenvironment in infinitesimally small quantity and participate in the regulation of gene expression in the presence or absence of appropriate signals. DNA binding and transactivation domains are found to be the two most exclusive parts of any TF. Besides that, TFs often possess domains responsible for interacting with proteins to form homo and heterodimers. Most of the mammalian TFs are involved in the regulation of multiple genes and these diverse functions are determined by their interactions with cofactors associated with the pathway of gene regulation. Broadly, mammalian TFs are classified based on the signature domains such as zinc finger, basic helix-loop-helix (bHLH), basic leucine zipper (bZIP) domain, and each signature domain plays a key role in determining the three-dimensional structure of the protein associated with the functional activities. The expression and functional activities of TFs are tightly coupled with cell proliferation, differentiation, and apoptosis. The uncontrolled expression or functional abnormalities of TFs leads to the initiation or establishment of diseases like cancer. Almost 300 TFs and associated cofactors are identified, based on the analysis of cancer genes, and considered as a major contributor to the development of various types of cancers [1].

Developing novel therapeutics in cancer is continuously evolving and potential novel targets are emerging routinely with the intention to reduce the mortality and the suffering of cancer patients. The extreme adaptive power of cancer cells, generations of secondary tumours through metastasis and developing drug resistance during or after responding to the primary therapy pose major challenges to researchers in controlling the disease progression and subsequently the saving of lives.

Development of cancer is strongly related to genetic abnormalities including TFs [2]. Along with that, the genetic diversities associated with each type of cancer are considered i) as a major contributing factor to introduce further complexities to the disease and ii) proposed to be the most rate-limiting factor in developing a universal treatment. So, identification, as well as validation of novel targets, is the continuous underlying process in cancer drug discovery.

Perhaps the most fundamental part of the identification of a new target is to understand the regulatory mechanisms and the key players that control the proliferation of cancer cells. Over the last few decades, researchers have applied several cutting-edge cells and molecular biology techniques to reveal the network and pathways of the regulatory mechanisms that drive the uncontrolled proliferation of cancer cells. As a result, we have more comprehensive pictures of the several regulatory networks than ever before that control the gene expression, signal transductions, and several other fundamental cellular processes directly or indirectly associated with the proliferation and differentiation of mammalian cells. A detailed fine print of these regulatory networks is a cornerstone for the identification of novel targets in drug discovery. One of the most fundamental questions in any form of drug discovery is the identification and validation of targets. Particularly in the case of cancer, it is more complicated because targets are predominantly endogenous biomolecules engaged with many essential cellular activities. Cancer cell proliferation, invasion, metastasis, angiogenesis, and apoptosis are regulated by multiple hyperactive and/or modulated cellular networks that include extracellular signaling networks, transmembrane receptors, intracellular protein kinases, and TFs [3]. It is conceivable that these vast cellular networks can potentially generate several targets that could lead to the development of novel therapeutics. On the other hand, functional inactivation or selective degradation of those molecules would likely lead to severe side effects.

Several biomolecules such as enzymes, receptors, membrane transporter, and TFs are considered as potential targets in drug discovery. However, the evidence is in the favour of the fact that some biomolecules are considered as better targets than others though they are potentially very similar. For example, enzymes are considered as a more-preferred target than TFs, mainly because many successes were reported in manipulating the activities of an enzyme by designing an inhibitor rather modulating the activities of a TF. For example, phospholipase C (PLC) is one of the enzymes that work in coordination with other signal transduction pathways such as phosphatidyl inositol-3 kinase (PI3 kinase), myosin activated protein kinase (MAP kinase) to control cell proliferation, differentiation, and angiogenesis. Among the six isozymes of PLC, PLC-γ1 is one of the most studied enzymes, which plays a major role in tumorigenesis [4] and became an attractive target for cancer treatment. Several small-molecule inhibitors (U-73122, D-609, caloporoside), capable of modulating the activity of PLC-γ1, were developed and shown to demonstrate anticancer activity [5].

In somatic cells, gene expression is controlled through the modification of histones via acetylation and de-acetylation by the group of enzymes known as histone acetyltransferases (HAT) and histone deacetylases (HDAC). The activities of HATs and HDACs are found to be unbalanced in cancer cells. Depending upon the types of cancer, HDAC inhibitors have shown to induce cell cycle arrest, differentiation, and reduced angiogenesis. HDAC inhibitor vorinostat, romidepsin, and panobinostat are currently approved for the treatment of T-Cell lymphoma and multiple myeloma [6]. On the other hand, over the last few decades, several hundred TFs were identified and their unparalleled roles in the proliferation and differentiation of mammalian cells were very well established. However, the failure rates in drug development are overwhelmingly high when TFs’ are targeted.

At the beginning of the era of targeted drug discovery, NF-κB and p53 were considered as the two very promising candidates due to their critical, multifunctional roles in several cellular activities as well as in the development of tumorigenesis. Till today, close to eight hundred inhibitors of NF-κB have been developed capable of inhibiting DNA binding activity, transactivation, and the expression of this TF. However, none of these inhibitors were found to be clinically effective [7, 8].

Since p53 is a tumour suppressor, therefore, the reactivation of this protein in cancer cells were proposed to be effective in controlling the proliferation of cancer cell or tumour. Activation of p53 is coupled with the dissociation of this protein with another protein, mouse double minute 2 homolog (MDM2). Small molecule inhibitors such as nutlins, spirooxindoles, benzodiazepinediones, and piperidinones found to be very effective in blocking p53-MDM2 interaction, but in clinical trials, patients with liposarcoma and leukaemia, these inhibitors generated severe side effects and they were withdrawn from the trials [9]. Observations, like this, were also reported when targeting other TFs and therefore, considered as major contributing factors in not considering them as priority targets. However, recent research is successful in shedding some light on this problem and thus this review aims to revisit the strategies of TF-targeting based on our updated knowledge of drug discovery in this area.

Many avenues of targeting TFs have emerged recently [10] which may be broadly categorized into four sections such as i) targeting regulatory mechanisms that control the gene expression, ii) inhibiting the DNA binding domain, iii) blocking the functional association with its co-factors, and iv) selective degradation of proteins.

## Targeting the regulatory mechanisms that control the gene expression

Increased expression of TFs is observed to be associated with the development and maintenance of several malignant cancers. In many cases, this elevated level of expression is due to upregulated transcription activity or due to the binding of *cis*- or *trans*-acting factors to the regulatory element of the gene that encodes the TF. In other cases, it is activated by a new recombinant transcriptional activator generated through chromosomal aberrations. Such recombinant transcription super activators modify the expression of several genes related to cell proliferation. Significant examples of this kind of transcriptional upregulations or miss regulations [11] have been studied recently in great detail and a few of them will be considered in the discussion.

**Figure 1.**
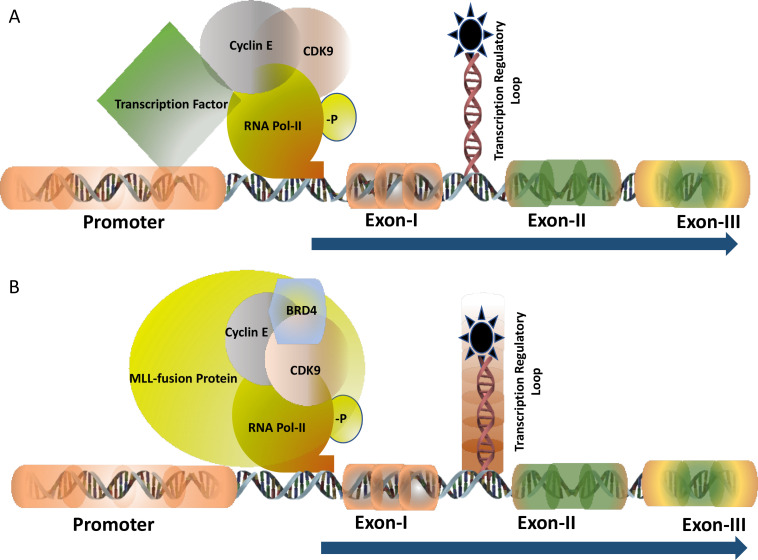
TF of c-MYB: transcription of c-MYB is regulated by the transcription pausing mechanism where the pausing site is located between Exon-I and Exon-II (shown as a loop). Panel A shows the transcription is regulated at the pausing site and driven by the gene-specific TF with the recruitment of CyclinT1 and CDK9 followed by the phosphorylation of RNA Pol-II (-P indicated it is phosphorylated). Panel B demonstrates the transcription is regulated by the MLL-fusion protein in leukaemia where BRD4 is also recruited into the protein complex besides CyclinT1 and CDK9. The regulatory pausing site is functionally inactive (indicated by the shed) causing unregulated transcription

TF c-MYB activates many cell proliferation specific genes. The half-life of the c-MYB mRNA in the somatic cells is very short indicating that the transcription is highly regulated at a particular stage of the cell cycle. In human breast epithelial cells, *c-Myb* expression is regulated by estrogen receptors (ERs) [12]. Analysis of breast tumours samples indicated a very high level of expression of estrogen in postmenopausal women in comparison with their pre-menopausal counterparts though, the expression of ERs remains unchanged [13]. This high level of estrogen can activate the expression of c-MYB which in turn drives uncontrolled cell proliferation by activating one of its downstream anti-apoptotic genes *Bcl2*. Several *in vitro* and *in vivo* studies, carried out by our group, clearly demonstrated that the c-MYB expression is absolutely required for the proliferation of breast cancer. Therefore, downregulation of ER activities with an anti-estrogen drug like tamoxifen is considered to be one of the accepted ways of treating estrogen-positive breast cancer. However, the laboratory of ours and others investigated to develop an alternative therapeutic model in estrogen-positive breast cancer because more than one-third of breast cancer patients develop resistance to anti-estrogen therapy during the course of treatment [14]. Our analysis, at the molecular level, identified that ERs need to be associated with cyclin-dependent kinase 9 (CDK9)-a protein kinase which is a part of the positive transcription elongation factor b (PTEF-b) complex required for post-transcriptional regulation of viral as well mammalian genes (Figure 1A). Estrogen positive breast cancer cells were found to be very sensitive to CDK9 inhibitors and at a sub-nanomolar concentration of CDK9 inhibitor, the cell undergoes apoptosis associated with a several-fold downregulation of c-MYB expression. However, this effect of CDK9 inhibitor can be overcome by ectopic expression of c-MYB in breast cancer cells [15, 16] establishing the fact that the c-MYB expression is the primary target of these inhibitors. CDK9 inhibitors are currently in clinical trials such as in acute myeloid leukaemia [17] but it has never been tested in breast cancer. This is currently an open area where more investigations are required to validate this model *in vivo* followed by clinical trials.

The mixed lineage leukemia (MLL)-fusion protein leukaemia is considered one of the most aggressive forms of diseases where the mortality rate is as high as 50%. Mixed lineage leukemia protein-1 or MLL-1 has histone H3 lysine 4 (H3K4) methyltransferase activity and form almost 70-inframe fusion proteins with several oncogenic partners through reciprocal chromosomal translocation. The fusion protein retains the N-terminal DNA domain of MLL protein and the conserved domain to interact with other co-factors such as multiple endocrine neoplasia type 1 (MEN-1). The c-terminal domain is contributed by the transactivation domain of several partner proteins such as AF9, ENL, ELL, AF4, and many others. MLL-fusion proteins form a multiprotein transcription super elongation complex that recruits DOTL1, BRD4, and PTEF-b through interaction with the transactivation domain [18]. Expression of *c-Myb* is regulated by the MLL-fusion protein at the transcriptional level and this persistence high-level expression is essential for the proliferation of the leukemic cells (Figure 1B). Therefore, c-MYB is considered as a potential therapeutic target in leukaemia. Inhibitors of DOTL1, BRD4, and PTEF-b (CDK9 inhibitor) showed very promising results both *in vitro* and *in vivo* in MLL-fusion protein leukaemia [19]. For example, the DOTL1 inhibitor pinometostat (EPZ-5676) inhibits the histone methyltransferase activity and found to be very specific and effective to inhibit leukaemia in animal models. A detailed study in our laboratory showed that CDK9 inhibitors block MLL-AF9 mediated activation of *c-Myb* and inhibition of the proliferation of leukemic cells [20]. The phase-I clinical trial with pinimetostat showed promising results amid some side effects [21]. Similarly, numerous BRD4 inhibitors such as mivebresib (ABBV-075), BMS-986158 are currently in clinical trials in haematological cancer and solid tumours [22, 23]. Therefore, targeting the regulatory mechanism of the TFs for finding a novel drug target is continuously expanding and it is expected more specific drugs with minimal side effects will be added successfully to the treatment regimen soon.

Mammalian genes are regulated at the transcriptional and epigenetic levels. While TFs are associated with the transcriptional control mechanism, epigenetic regulation is associated with the accessibility of the chromosome through the modification of histones required to make a complex 3-D structure of the chromosomes. Genes encoding the TFs’ are also regulated epigenetically and it has been demonstrated that overexpression of oncogenic TFs is a result of permanent epigenetic modification of genes. Therefore, it is expected that drugs targeting the enzymes responsible for epigenetic modification would affect the expression of TF’s. However, the most challenging part here is the identification of a particular enzyme modifying histone because these histone-modifying enzymes are working simultaneously to control the expression of many genes and interruptions would generate severe off-target effects. The epigenetic regulation of the oncogenic TF MYC is one of the well-studied models. In case of MYC, this study had additional significance because this TF lacks a defined targetable domain. Inhibitors of histone-modifying enzymes such as HDAC, DNA methyltransferases, and bromodomain and extra-terminal motif (BET) were used to determine the effect on transcription. One of the BET family members, BRD4 recruits P-TEFb (a heterodimer of Cyclin T1 and CDK9) which is required for its functional activities. Inhibitors of BRD4 showed its reduced binding at acetylated histones located in the promoter and the enhancer region of the *MYC* gene and downregulates its expression. The BET inhibitor OTX015/MK-8628 which interferes with its binding with acetylated histones was used to conduct phase-I and II clinical trials, however, it was not successful due to severe side effect. Another BET inhibitor BI 894999 is now in clinical trials in combination with the inhibitor of P-TEFb. This combination treatment demonstrated a synergistic effect in the inhibition of cancer cell proliferation and a reduction of tumour growth [24, 25].

## Inhibiting the DNA binding domain TF’s

Every TF is designed to bind a specific DNA sequence and a defined conformation is required to recognize the stretch of DNA harbouring the specific binding sequence. Therefore, studying the structure of the DNA binding domain and designing a drug that could block this domain is one of the established approaches to the targeted drug discovery. X-ray crystallography plays a very important role in this area because it provides very fine molecular details (at the atomic level) and therefore could predict the shape of a possible inhibitory molecule that would block the DNA binding cavity. Using the computer-assisted drug discovery (CADD), which utilizes the crystallographic structure of the protein or specific domain of the protein, it is possible to run a virtual screening of chemical libraries to make a list of plausible ‘hits’ for carrying out *in vitro* and *in vivo* assays. For our better understanding, we would like to mention very briefly about a few recent approaches in this area.

In prostate cancer, human androgen receptors (ARs), a TF, are responsible for the development and progression of the disease. Enzalutamide is a small molecule inhibitor that blocks the ligand-binding domain of this receptor to inactivate the transcriptional activities. However, resistance to this drug was also reported after treating patients for a while. In a CADD approach, Li et al. [26], performed a virtual screening using the DNA binding domain of the AR and identified a synthetic analog of the original of the anticancer drug morpholine. It was also noticed that the efficiency of this drug is very much comparable with Enzalutamide. Further studies of Li et al. [26], also showed that this new drug can be applied to the patients resistant to the Enzalutamide treatment.

Constitutive activation of the TF signal transducer and activator of transcription 3 (STAT3) has been shown to be essential for the aggressiveness of the malignant tumours and thus making this TF as an attractive target for drug discovery. The STAT3 protein has an Src homology 2 (SH2) domain at the c-terminal region of this protein and a series of drugs targeted to this domain was found to be unsuccessful in clinical trials. In their study, Huang et al. [27], targeted the DNA binding domain of this protein and identified a small molecule inhibitor S3-54A18 following an *in silico* approach and found this novel inhibitor to be effective to block the DNA binding of this protein as well as stops the tumour growth and metastasis *in vivo*. In both of these studies mentioned here, all initial approaches were to target other novel domains (SH2 for STAT3 and ligand-binding domain of the AR) but not the DNA binding domain due to the undruggable nature of this domain. However, several biophysical methods, X-ray crystallography, CADD, and other *in silico* approaches are currently working in collaboration to target the DNA binding domain of TF’s which were almost inaccessible in the early days of drug discovery.

Based on the nature of the DNA binding domain, TFs are also classified as zinc finger, bHLH, bZIP group of proteins. The largest family of human TF belongs to the zinc finger group proteins. By rearranging the zinc finger domains, which are also associated with the change in the conformation of the protein, these groups of TFs are capable of binding promoters of multiple genes. This phenomenon of conformational modifications is also applicable for the b-HLH and b-ZIP group of protein due to the flexibility of the DNA binding motif. Targeting the DNA binding domain of these groups of proteins is more challenging because of the high risk of generating off-target effects. For example, currently, our knowledge is very much limited about targeting a specific zinc finger a protein with multiple zinc finger motif. Since zinc fingers are capable to bind other divalent metal ions, therefore, attempts to add other metal ions such as Co (II), Ni (II), and Cu (II) in excess found to be effective to reduce the DNA binding activity of the zinc finger proteins [28]. However, the toxic effect of the metal ions would be a major bottleneck for the therapeutic application. Therefore, more research is required to overcome the challenges of accurately targeting the DNA binding domains of TF’s belong to these categories.

## Blocking the functional association of TF’s with its co-factors

TFs maintain a dynamic equilibrium between their DNA bound and unbound form. This equilibrium is necessary for the genes that are expressed constitutively at a very low level. However, for the inducible gene expression, the binding of a set of specific TFs along with cofactors is upregulated in the presence of appropriate signals. Therefore, the direct or indirect association of TF and its cofactors has been found to be essential to trigger the active transcription. On the other hand, interference of this association, if possible, would downregulate the transcription and expression of genes even in the presence of appropriate signals. Over the last few decades, several such interactions have been documented and the importance of these interactions was found to be very critical in controlling gene transcription. However, successful attempts of targeting these interactions in drug discovery are not at all very impressive which is perhaps due to the very complex nature of these interactions that prevent in designing or identifying appropriate molecule(s) capable of inhibiting such interactions.

**Figure 2.**
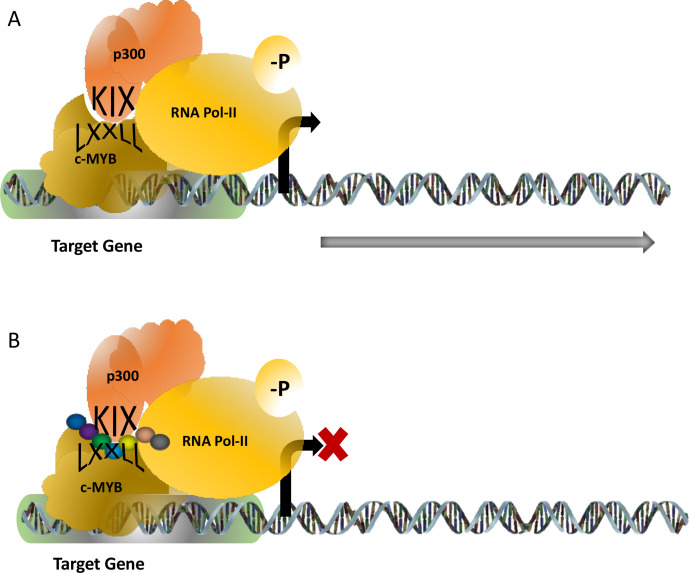
c-MYB-p300 interaction activates target genes required for the proliferation of leukaemia. The interaction is mediated through the KIX domain of p300 and the LXXLL motif of c-MYB (panel). Attempts to design synthetic peptides (shown as a string of beads) were found to be successful to interrupt this interaction

TF c-MYB plays a key role in haematopoiesis and in the development and maintenance of leukaemia. For its functional activity, c-MYB associates and physically interacts with many proteins and among the several proteins, the p300-c-MYB interaction is one of the most studied interactions because it is implicated as a major driver for the leukemic transformation (Figure 2). The interaction of p300 (which has histone acetyltransferase activity), and c-MYB is mediated through the highly conserved KIX (kinase-inducible domain interacting domain) domain of p300 and the conserve LXXLL motif of c-MYB located in the transactivation domain. Mutations at the LXXLL motif prevents this interaction and the activity of the c-MYB by modulating its DNA binding capacity. Our laboratory worked on a transgenic mouse model where this interaction is abrogated and observed that this transgenic mouse failed to develop leukaemia upon expression of oncogenic fusion proteins such as acute myeloid leukemia-eight-twenty-one (AML-ETO) and MLL-AF9 [29]. Therefore, it appeared that this interaction is a valid target to functionally inactivate c-MYB in leukaemia [30]. A few attempts were made recently to disrupt this interaction and one of them was reported by Uttarkar et al [31]. In their studies, a derivative of naphthoquinones, Plumbagin, was shown to downregulate the c-MYB function by inhibiting this interaction and this drug also prevented the proliferation of AML cells without affecting the normal haematopoietic progenitor cells. Plumbagin is known for its anticancer activities for a while however no human clinical trials have been reported till today and the mechanism of action of this drug was not very well understood. Our laboratory designed several short peptides with modified amino acids targeting the KIX domain and a few of them were capable of interrupting the interaction as revealed by our cell-based assay system.

Several examples of active interaction, like above, are already known to be functionally important for the disease development, however, very few of them were moved forward in the pathway of drug discovery. For example, oncogenic TF MYC interacts with WDR5-a protein with druggable pockets, for the activation of genes. This interaction is structurally very well defined based on the X-ray crystallography data and is responsible for leukemogenesis. However, no attempts were reported so far as to target this interaction [32]. Therefore, despite having significant potential, the interaction of TF and cofactor did not proceed as it was expected for the drug development.

Besides interacting with its co-factors, TF’s often from homodimers such as STAT3, hypoxia-inducible factor 1 (HIF-1) and this homodimerization are pivotal for the functional activity of this protein. As it was mentioned previously, STAT3 drew major attention recently because it is constitutively expressed in several cancers. The dimerization occurs through the reciprocal interaction of the conserved tyrosine residue when phosphorylated at the SH2 domain [33]. Over the last few years, Mandal et al. [34–36], at the MD Anderson Cancer Center, TX, USA studied extensively in this field and developed selective peptide mimetics and their derivatives that can block the phosphorylation of the tyrosine residues that are critical for dimerization. These peptides are very selective to inhibit the activities of STAT3 but not the STAT1 or 2 and prevent the growth of cancer cells harbouring the constitutive expression of STAT3. With such promising results, those peptidomimetic inhibitors are currently at the very basic level of drug development.

HIF-1 is a bHLH TF considered as another promising target. It plays a very crucial role in the expression of genes related to the angiogenesis and controls the expression of vascular endothelial growth factor which is upregulated to several folds during tumour growth. HIF1α is required to form a heterodimer with HIF1β for the activation of its target genes. Since HIF1 is an appealing target, several inhibitors were identified targeting this TF such as DNA binding activity, degradations, and heterodimer formation. A high throughput genetic screening was conducted by Miranda et al. [37], to identify a cyclopeptide to block the heterodimer formation of HIF1. The reported cyclo-CLLFVY was a cyclic peptide capable of blocking the heterodimerization and reduced the hypoxia-mediated signal in several cancer cell lines.

## Selective degradation of the TF overexpressed in cancer cells

Most of the TF in somatic cells express at a very low level and this low basal level of expression is required to bind the high-affinity DNA binding site. However, under the condition of elevated level of expression, TFs bind to the low-affinity binding site of the DNA and can activate several genes [38]. Often this low-affinity binding can activate enhancers located several kilobases up or downstream of the gene and can drive uncontrolled gene expression. Therefore, one of the proposed models of targeting TFs is to accelerate the *de novo* degradation of these proteins. Mammalian cells have endogenous protein degradation pathways where proteins are degraded followed by ubiquitination and sumoylation. The anti-infective drug mebendazole exhibits anti-tumor activities by reducing cell proliferation and enhancing apoptosis mediated through the activation of the protein degradation pathway. In one of such recent efforts, Walf-Vorderwülbecke et al. [39], showed that mebendazole treatment reduced the proliferation of AML cells by facilitating the degradation of c-MYB. Recently, targeted protein degradation technology has been introduced with significant therapeutic potential. Chimeric molecules such as proteolysis targeting chimeras (PROTACs) and specific and non-genetic IAP-dependent protein erasers (SNIPERs) were developed for facilitating the degradation of target proteins. Both chimeric molecules were demonstrated to be efficient at a sub-nanomolar level in the xenograft model with antitumor activity and currently, they are in the early phase of clinical trials [40].

The purpose of this review is to discuss several strategies to target TFs as a part of targeted drug discovery. However, promising results were reported where activities of TFs were modulated by potential drug candidates and the mechanism of action is not clearly understood. For example, several natural bioactive molecules have been found to be modulating the activity of the signaling pathway connected to the TF AP-1 [41]. Similarly, several inhibitors such as caryophyllene oxide, garcinol obtained from natural sources found to be active in blocking STAT3, by targeting signal transduction pathway connected to this TF. These molecules were shown to be very effective in controlling the growth of tumour in animal model [42] and expected to be added to the treatment regimen in the near future [43–45]. The major TF’s included in this article and their broad-spectrum roles in various aspects in cancer development is summarized in the Table 1.

**Table 1.** TFs and their role in cancer

**Name**	**Description**	**Role in cancer**
NF-κB	A dimeric TF that combines NF-κB and Rel protein. It regulates the expression of several genes related to inflammation, innate and adaptive immunity and stress response.	It plays an important role in tumorigenesis, inflammation, preventing apoptosis, supporting angiogenesis and metastasis.
p53	Also known as TP53 functions as TF when forms a tetramer. It plays important role in DNA damage, cell-cycle regulation and apoptosis.	It acts as a tumour suppressor. Most tumours have mutations in the *p53* gene. Mutated proteins cannot bind DNA effectively and cells lose their control on cell cycle regulation and apoptosis.
c-Myb	As an oncogenic TF, it controls the several genes related to cell proliferation, differentiation and apoptosis. It plays a key role in haematopoietic cell proliferation and lineage differentiation.	Over expression of c-Myb is noticed in breast, colon and haematopoietic cancer. In solid tumours, c-Myb is required for proliferation and in leukaemia, c-Myb controls several downstream genes necessary to maintain the proliferation of leukaemic cells.
MLL-AF9	A chimeric TF formed by chromosomal translocation of the N-terminal DNA binding part of the MLL protein (lysine-specific methyltransferase 2A located in chromosome 11) with the c-terminal part of AF9 protein (gene is located in chromosome 9). This TF can bind promoters of a wide range of proteins as well as the enhancer elements.	The *RUNX1* gene (runt-related TF1) is one of the most well documented downstream targets of MLL-AF9. RUNX1 is a TF that controls granulocytic differentiation. MLL-AF9 driven overexpression of RUNX1 mediates leukemogenic transformation.
c-MYC	Oncogenic TF which is responsible to control cell proliferation and apoptosis.	Overexpression of c-MYC is observed in more than 40% of tumours. Overexpression caused by gene amplification deregulates the cell proliferation and apoptosis pathways.
STAT3	A master TF that controls the expression of several genes related to the innate and adaptive immunity. It plays an integral part to transduce the signal from receptors to the transduction factors to relocate in the nucleus.	It acts as a key player in supporting tumour microenvironment which includes maintaining hypoxic condition, blood vessels and extracellular matrix (ECM) formation, immune cells, and inflammatory cells proliferation.
AML1-ETO	A fusion protein generated by chromosomal translocation in AML. The fusion protein comprises of conserved runt homology (which is the DNA binding domain) from the hematopoietic TF RUNX1 (also known as AML1) and ETO repressor protein. It is considered as a transcriptional repressor of all RUNX1 target genes.	AML1 acts as a transcriptional activator but this fusion protein acts as a transcriptional repressor in granulocytic differentiation and drives granulocytes in the mode of continuous uncontrolled proliferation.
HIF-1	A key TF that regulates the physiological response to the low oxygen concentration or in hypoxia.	It plays a crucial role in maintaining the hypoxic tumour microenvironment by regulating several genes related to this phenomenon. Elevated expression of this TF is associated with poor prognosis and high metastasis.
AP1	It forms a heterodimer with the oncoproteins c-FOS or c-JUN and regulates genes related to the cell proliferation, differentiation, apoptosis and angiogenesis.	It acts as an oncogenic factor or tumour suppressor depending on the nature of the cell types, stage of the tumour and its genotypes.

## Conclusion

The effect TF on gene expression and the development of diseases are a very well-established phenomenon. It is unconditionally accepted that functional inactivation or downregulation of the overexpressed TF would generate an immediate effect on cellular and metabolic processes. However, in the field of drug discovery, TFs were sitting at the ‘back seat’ as a target for a long period of time because of the undruggable nature of these molecules. Over the last few decades, several new technologies were applied to explore this undruggable nature and as a result, some promising new candidates targeting cofactors, or the TFs itself were identified, and they are currently in the different phases of human clinical trials. It is also well understood that targeting TF is still a very high-risk area of targeted drug discovery. Therefore, to increase the rate of success, an extensive search is necessary to identify a panel of potential drug candidates in the early stage of drug discovery to obtain a clinically potential molecule(s) with modest adverse effects. If we are successful, then drugs targeting TF would be a new armamentarium for cancer treatment.

## Abbreviations

AML: acute myeloid leukemia
AR: androgen receptors
BET: bromodomain and extra-terminal motif
bHLH: basic helix-loop-helix
bZIP: basic leucine zipper
CADD: computer-assisted drug discovery
CDK9: cyclin-dependent kinase 9
ERs: estrogen receptors
HAT: histone acetyltransferases
HDAC: histone deacetylases
HIF-1: hypoxia-inducible factor 1
MDM2: mouse double minute 2 homolog
MLL: mixed lineage leukemia
PLC: phospholipase C
PTEF-b: positive transcription elongation factor b
SH2: Src homology 2
STAT3: signal transducer and activator of transcription 3
TF: transcription factor

## Declarations

### Copyright

© The Author(s) 2020.
